# Monalysin, a Novel *ß*-Pore-Forming Toxin from the *Drosophila* Pathogen *Pseudomonas entomophila,* Contributes to Host Intestinal Damage and Lethality

**DOI:** 10.1371/journal.ppat.1002259

**Published:** 2011-09-29

**Authors:** Onya Opota, Isabelle Vallet-Gély, Renaud Vincentelli, Christine Kellenberger, Ioan Iacovache, Manuel Rodrigo Gonzalez, Alain Roussel, Françoise-Gisou van der Goot, Bruno Lemaitre

**Affiliations:** 1 Global Health Institute, Ecole Polytechnique Fédérale Lausanne (EPFL), Lausanne, Switzerland; 2 Centre de Génétique Moléculaire, CNRS, Gif-sur-Yvette, France; 3 Structural Immunology, AFMB UMR 6098 CNRS/UI/UII, Case 932, Marseille, France; Stanford University, United States of America

## Abstract

*Pseudomonas entomophila* is an entomopathogenic bacterium that infects and kills *Drosophila. P. entomophila* pathogenicity is linked to its ability to cause irreversible damages to the *Drosophila* gut, preventing epithelium renewal and repair. Here we report the identification of a novel pore-forming toxin (PFT), Monalysin, which contributes to the virulence of *P. entomophila* against *Drosophila*. Our data show that Monalysin requires N-terminal cleavage to become fully active, forms oligomers in vitro, and induces pore-formation in artificial lipid membranes. The prediction of the secondary structure of the membrane-spanning domain indicates that Monalysin is a PFT of the ß-type. The expression of Monalysin is regulated by both the GacS/GacA two-component system and the Pvf regulator, two signaling systems that control *P. entomophila* pathogenicity. In addition, AprA, a metallo-protease secreted by *P. entomophila*, can induce the rapid cleavage of pro-Monalysin into its active form. Reduced cell death is observed upon infection with a mutant deficient in Monalysin production showing that Monalysin plays a role in *P. entomophila* ability to induce intestinal cell damages, which is consistent with its activity as a PFT. Our study together with the well-established action of *Bacillus thuringiensis* Cry toxins suggests that production of PFTs is a common strategy of entomopathogens to disrupt insect gut homeostasis.

## Author Summary

Insects are potential reservoirs for microbes and ideal vectors for their transmission due to their motility and capacity to live in bacteria-rich environments. This is exemplified by fruit flies that live in rotting fruits and are capable of transmitting phytopathogenic bacteria. Insects are notably resistant to microbial infection allowing them to colonize these microbe-rich environments. To study how pathogenic bacteria disrupt gut homeostasis, we investigated the interactions between *Drosophila* and a newly identified entomopathogen, *Pseudomonas entomophila*. Ingestion of *P. entomophila* inflicts severe damage to the *Drosophila* intestine. How damages are inflicted, however, remains unknown. In this study, we identified a secreted protein that plays an important role in the damage inflicted by *P. entomophila* to the *Drosophila* gut. We showed that this protein is a pore-forming toxin (PFT) that we named Monalysin. Our study reveals that Monalysin oligomerizes into ring-like structures that form pores into the plasma membrane of target cells leading to the disruption of membrane permeability and cell death. Our work together with studies on the insecticidal Cry toxins produced by *Bacillus thuringiensis* suggests that production of PFTs is a common strategy of entomopathogenic bacteria to interfere with insect gut homeostasis.

## Introduction

The intestinal epithelium has a role in defining the barrier between the host and the external environment [1]. This barrier protects the host against invasion and systemic dissemination of both pathogenic and commensal microorganisms. Both resistance and tolerance mechanisms contribute to maintain the gut integrity from the assault of infectious bacteria [2]. Resistance mechanisms involve the activation of various local immune responses that directly target pathogens. In contrast, tolerance mechanisms involve the activation of repair and stress pathways that quickly seal damages caused by infectious agents. Pathogenic bacteria have the capacity to overcome gut defenses and impede the return to homeostasis [3]. To study how pathogenic bacteria disrupt gut homeostasis, we chose to investigate the interactions between *Drosophila* and a newly identified entomopathogen, *Pseudomonas entomophila*. *P. entomophila* is closely related to the saprophytic soil bacterium *Pseudomonas putida* [4], [5]. It was originally isolated from a fly sampled in Guadeloupe and subsequently shown to be lethal to *Drosophila* larvae and adults after ingestion. *P. entomophila* can also effectively kill members of other insect orders (e.g. *Bombyx mori*, *Anopheles gambiae, Galleria mellonella*). After ingestion, *P. entomophila* is able to persist in the *Drosophila* gut. It induces the expression of antimicrobial peptide genes via the Imd pathway, both locally in the intestinal epithelium and systemically in the fat body, an organ analog to the mammalian liver [4]. It was shown that *P. entomophila* virulence is under the control of two global regulatory systems: the well known GacS/GacA two component system, and a second system involving a secreted secondary metabolite synthesized by the *pvf* gene products [4], [6]. The Gac system also controls the production of a secreted protease, AprA, which is important for *P. entomophila* to counteract the local immune response of *Drosophila* [7].

Recent studies revealed that upon bacterial infection, homeostasis in the gut is restored only when bacterial clearance is coordinated with the repair of infection-induced damage through epithelium renewal [8]–[10]. Epithelium renewal of the *Drosophila* gut is stimulated by the release of the secreted ligand Upd3 from damaged enterocytes, which then activates the JAK/STAT pathway in intestinal stem cells to promote both their division and differentiation, establishing a homeostatic regulatory loop [8], [9]. In contrast to infection with non-lethal bacteria, *P. entomophila* infection inflicts strong damage to its host without triggering an epithelial renewal [8], [11]. This suggests that the damages inflicted by *P. entomophila* are too severe to be repaired. How damages are inflicted however remains unknown. One hypothesis was that *P. entomophila* produces cytotoxic factors that damage the intestinal epithelium.

In this study, we identified a secreted protein that plays an important role in the damage inflicted by *P. entomophila* to the *Drosophila* gut. We showed that this protein is a pore-forming toxin (PFT) that we called Monalysin. Our work indicates that production of PFTs is a strategy used by entomopathogenic bacteria to disrupt gut homeostasis.

## Results

### Identification of a secreted protein involved in *P. entomophila* pathogenicity

**Figure 1:**
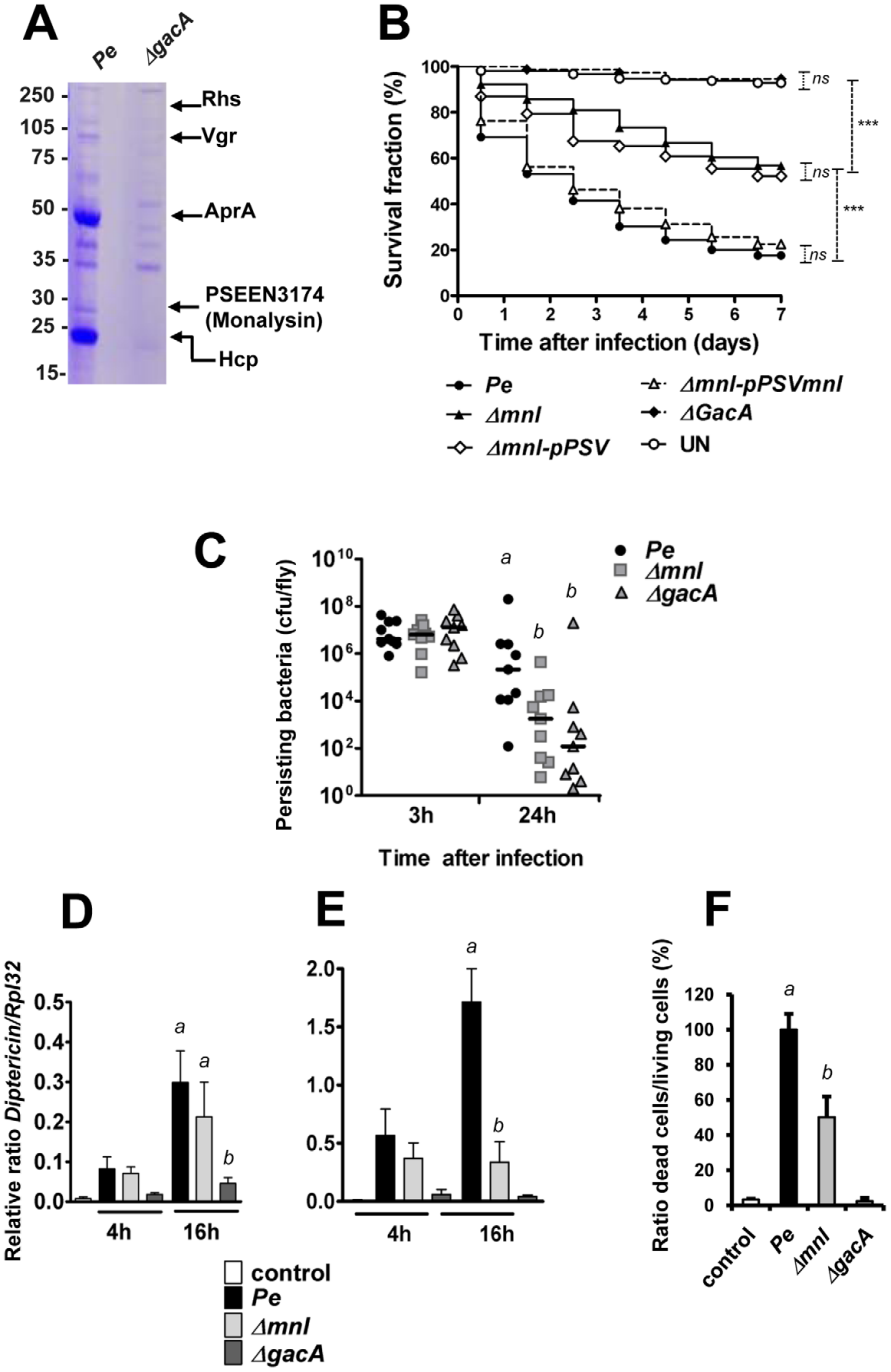
PSEEN3174 encodes a secreted protein, Monalysin, required for *P. entomophila* virulence. (**A**) SDS-PAGE analysis of culture supernatants from wild type *P. entomophila* and the *ΔgacA* derivative. Proteins extracted from culture supernatants were loaded on a SDS-PAGE and stained with coomassie blue. The nature of proteins identified by MALDI-TOF analysis of tryptic fragments is shown on the right. (**B**) Survival analysis of wild-type Oregon adult flies following infection by feeding with the *P. entomophila* wild-type strain (*Pe*), the *gacA*-deficient strain (*ΔgacA*), the *mnl* deficient strain (*Δmnl*) the *mnl*-deficient strain carrying a plasmid expressing a wild-type copy of the *monalysin* gene (*Δmnl-pPSVmnl*), or carrying the plasmid pPSV35 without any insert (*Δmnl-pPSV*). UN: unchallenged. The Kaplan-Meier log rank test was used to determine statistical significance. Dashed brackets represent the significance between the different infections (***: p<0.001, ns: not significant). (**C**) Bacterial persistence in wild-type Oregon flies as the number of colony-forming-unit (cfu) per fly. After infection by the *P. entomophila* wild-type strain (*Pe*), the *gacA*-deficient strain (*ΔgacA*), the *mnl* deficient strain (*Δmnl*), the number of cfu per fly was determined at the indicated time point. (**D and E** )Time-course analysis of *Diptericin* expression measured by RT-qPCR in (**D**) guts (local) or (**E**) whole flies (corresponding to the systemic fat body expression). (**F**) Cell death quantification using acridine orange staining. Results represent the percentage of dead cells (acridine orange positive nuclei) in the midguts of flies infected for 16 hours with the indicated bacterial strains. Results represent the average of four independent experiments. Statistical analysis was performed using a Wilcoxon test, and different letters indicate significantly different values (*P*<0.05).

We previously showed that *P. entomophila* secretes large amount of the metalloprotease, AprA, which can degrade antimicrobial peptides [7]. The production of this protease is regulated by the GacS-GasA system, known to control secondary metabolite production, protein secretion, and virulence determinants in γ-proteobacteria [12]. To identify additional factors responsible for *P. entomophila* virulence, we analyzed the culture supernatant of the wild-type bacterium and a *gacA* mutant by SDS-PAGE (**Figure 1A**). Bands corresponding to major secreted proteins in the wild type strain, but not in the *gacA* mutant were submitted to analysis by mass spectrometry. This allowed us to confirm that one of the major bands corresponds to the 51 kDa AprA. Three bands contained Hcp, Vgr and Rhs, proteins known to be secreted by the Type VI Secretion System (T6SS). T6SS are bacterial needle-like structure involved in the injection of effectors into the cytoplasm of eukaryotic but also prokaryotic cells [13]. We also identified a band with an apparent molecular weight of 30 kDa, containing a protein encoded by the uncharacterized gene *pseen3174* that we named Monalysin.

In order to investigate the role of these secreted proteins, we made a T6SS mutant (affecting the ORF *pseen0535*) and a *monalysin* (*mnl*) mutant (*Δmnl*), and tested their virulence in *Drosophila*. No difference could be observed between the wild type strain and the T6SS mutant. Interestingly, the *mnl* mutant presented a decreased pathogenicity. Indeed, survival analysis of *Drosophila* adults after oral infection with the wild-type strain, the *gacA* mutant, and the *mnl* mutant showed that only 40% of the flies infected with the *mnl* mutant succumbed within 3 days, while 70% of the flies died after infection with wild-type *P. entomophila* (**Figure 1B** **and Figure S6**). As previously shown [4], a *gacA* deficient mutant did not show any pathogenicity using this assay. The attenuated virulence of the *mnl* mutant was fully rescued by complementation with a wild-type copy of the *monalysin* gene.

### A mutant deficient in Monalysin production is affected in its abilities to induce cell damage in the *Drosophila* gut

It was previously shown that *P. entomophila* virulence towards *Drosophila* is associated to its ability to persist in the gut and the transcription of antibacterial peptide genes both locally and systematically [4]. In order to better characterize the role of the *monalysin* gene in the infectious process, we next compared the ability of the *mnl* mutant (*Δmnl*) to persist to that of the wild type strain or a *gacA* mutant. Flies were infected by feeding and bacterial loads were quantified at two time points **(** **Figure 1C** **)**. While bacterial loads were indistinguishable after 3 hrs, persistence of the *mnl* mutant and the *gacA* mutant were significantly decreased when compared to wild type bacteria [4]. We then compared the activation of the Imd pathway after infection by the wild type, the *gacA*, and the *mnl* mutant. We used reverse transcriptase quantitative PCR (RT-qPCR) to measure the expression of the *Diptericin* gene, a target of the Imd pathway, specifically in the gut (local response) or in whole flies (reflecting mostly the systemic expression of *Diptericin* by the fat body) (**Figure 1D and E**). As previously shown [7], *Diptericin* expression increased already 4 h after infection by *P. entomophila* and even more after 16 h, both in the gut and the fat body, an increase that was not observed for the *gacA* mutant [4]. The *mnl* mutant leads to an increase in *Diptericin* expression in the gut similar to that observed for the wild-type bacterium (**Figure 1D** **)**. However, while *Diptericin* expression increased to wild-type levels in the fat body 4 h after infection, no further increase was observed (16 h) in flies infected with the *mnl* mutant **(** **Figure 1E**).**

**Figure 2:**
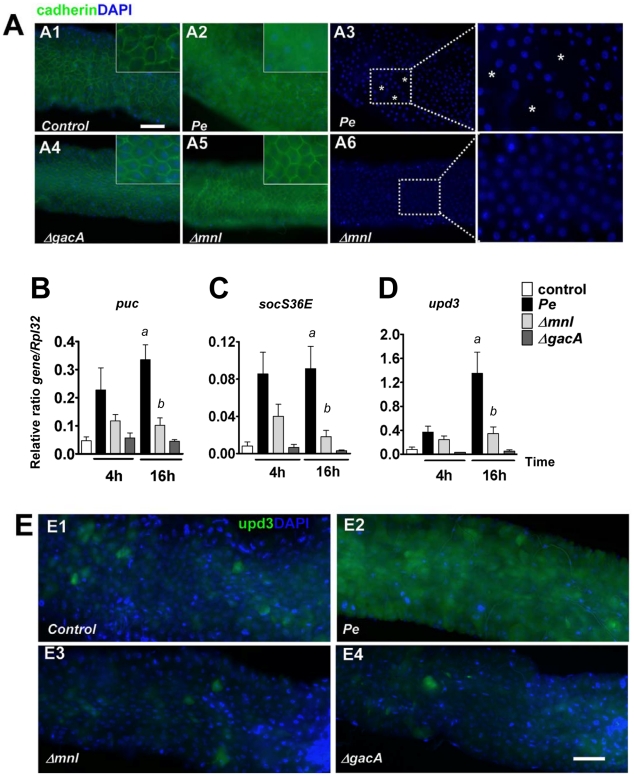
Monalysin contributes to *P. entomophila*-inflicted damage to the *Drosophila* gut. (**A**) Expression of the marker of adherens junction Cadherin-GFP 16 hours after infection with lethal doses of the indicated bacteria. Ingestion of wild type *P. entomophila* disrupts the pattern of Cadherin-GFP. **A3** and **A6** show DAPI of **A2** and **A5** respectively. The symbol (*) marks regions where DAPI staining is absent. (**B–D**) Analysis of *puckered* (*puc*), *unpaired3* (*upd3*), and *socs36E* expression measured by RT-qPCR in guts of infected flies. Statistical analysis was performed using a Wilcoxon test and letters indicate significantly different values (*P*<0.05). (**E**) Expression of the *upd3-Gal4, UAS-GFP* reporter in (**E1**) unchallenged flies or( **E2–E4**) 4 hours after infections with a sublethal dose of bacteria (OD_600_ = 10). In contrast to the wild type *P. entomophila* strain (**E2**), the *Δmnl* (**E3**) and the *ΔgacA* (**E4**) strains were unable to elicit upd3-GFP expression. Scale bars represent 50 µm.

We next investigated the contribution of Monalysin in the damage caused by *P. entomophila* to the *Drosophila* gut. We first monitor the induction of cell death upon bacterial ingestion using an acridine orange staining. A high number of dead cells were detected in guts from flies infected by wild type *P. entomophila*, but not in guts from flies infected by a *gacA* mutant as previously reported [6]. Interestingly, a reduced level of cell death was observed in the *mnl* mutant (**Figure 1F and S** **7**). Oral infection with *P. entomophila* resulted in a decrease of the adherens junction marker Cadherin-GFP (**Figure 2A**) and to morphologically altered guts, with regions devoid of enterocytes indicative of a disruption of tissue integrity (see the lack of nuclear DAPI staining due to the loss of cell in **Figure 2A** **3**). Interestingly, gut collected 16 hrs after oral infection with *gacA* and *mnl* mutants did not show any Cadherin-GFP signal decreases or a rupture of the gut integrity (**Figure 2A** **4 to 2A6**). Previous studies showed that ingestion of *P. entomophila* activates both JAK-STAT and the Jun N-terminal kinase (JNK) pathway in the *Drosophila* gut [8] that participate in the repair and stress responses, respectively [8], [14]–[16]. The activation of both pathways can be monitored by measuring by RT-qPCR the expression of *puckered* (*puc*) (a direct downstream target of JNK signaling) or *upd3* (a target of JAK-STAT signaling) and *Socs36E* (a target of JAK-STAT signaling that encodes a negative regulator of this pathway). **Figure 2B, 2C and 2D** shows that the *mnl* mutant was less efficient than wild type *P. entomophila* to activate the JNK and JAK-STAT pathways, yet more efficient than a *gacA* mutant. Consistent with the RT-qPCR analysis, expression of the *upd3-GFP* reporter gene (*upd3-Gal4, UAS-GFP*) was strongly induced in the gut of flies orally infected with a sub-lethal dose of *P. entomophila* but not the *mnl* mutant (**Figure 2E**). Altogether, these data show that even though the *mnl* mutant retains some ability to cause intestinal damage, this ability is strongly diminished compared to wild type *P. entomophila*. This suggested a specific role of the Monalysin protein in *P. entomophila* cytotoxicity towards *Drosophila*.

### Monalysin is a secreted cytotoxic protein

**Figure 3:**
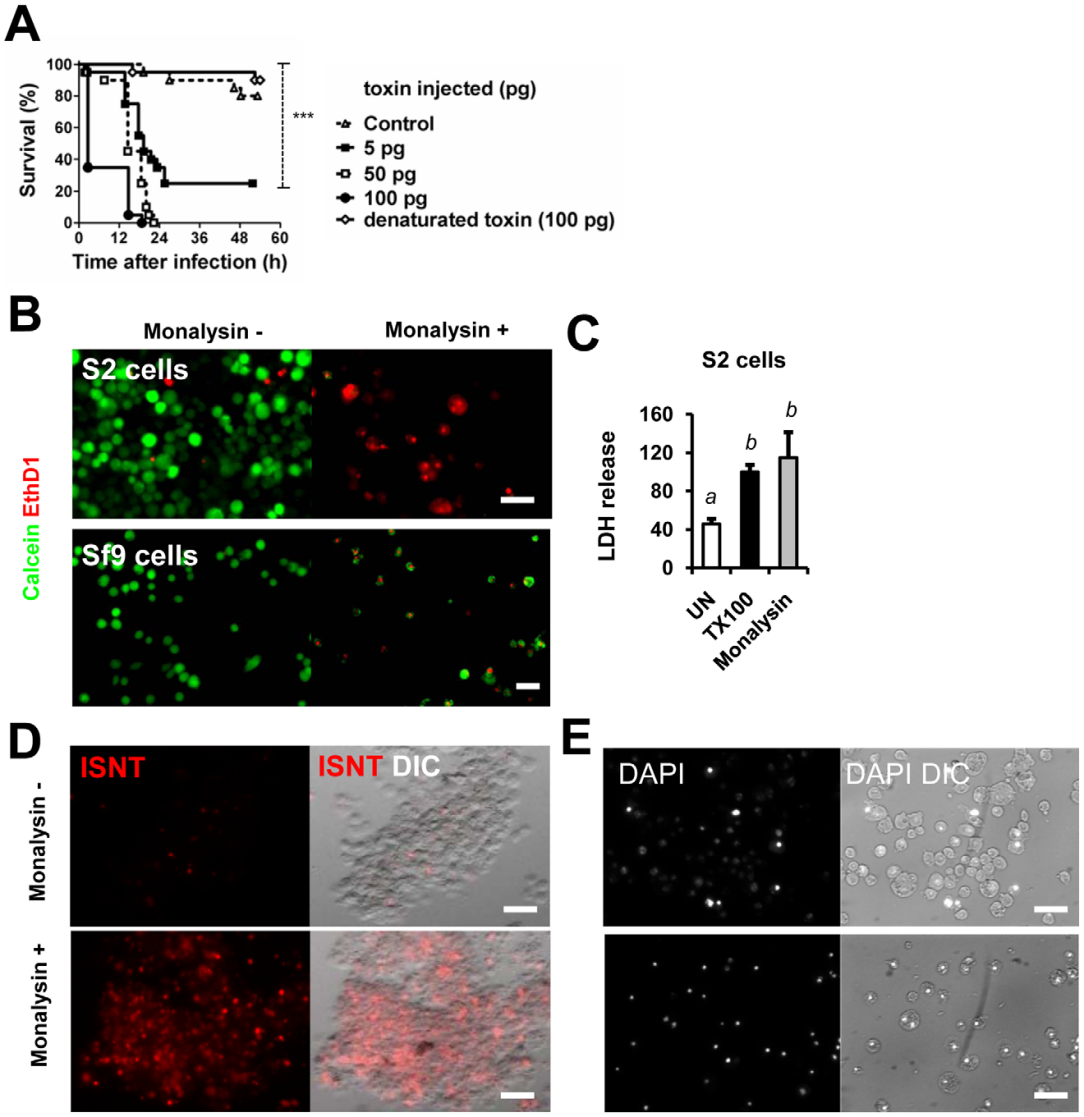
*Monalysin* encodes a cytotoxic protein secreted by *P. entomophila*. (**A**) Survival analysis of wild-type Oregon adult flies after injection of various quantities of Monalysin or heat-inactivated (denaturated) Monalysin. (**B and C**) Cytotoxic effect of Monalysin on insect culture cell line S2. (**B**) *Drosophila* S2 cells and *Spodoptera frugiperda* Sf9 cells were treated with Monalysin (Final concentration = 100nM) and stained with a live-dead viability reagent. Living cells are stained in green with Calcein while dead cells are stained in red with Ethidium homodimer 1 (EthD1, red). (**C**) The loss of viability was quantified by measuring the release of lactate dehydrogenase (LDH) from S2 cells. (**D**) DNA fragmentation in S2 cells was monitored by ISNT (*in situ nick translation).* (**E**) Chromatin condensation on untreated and Monalysin treated S2 cells was examined by DAPI staining. Phase-contrast and fluorescence views of the same microscopic fields are shown. (−) untreated cells, (+)  =  cells treated with Monalysin 100nM.

**Figure 4:**
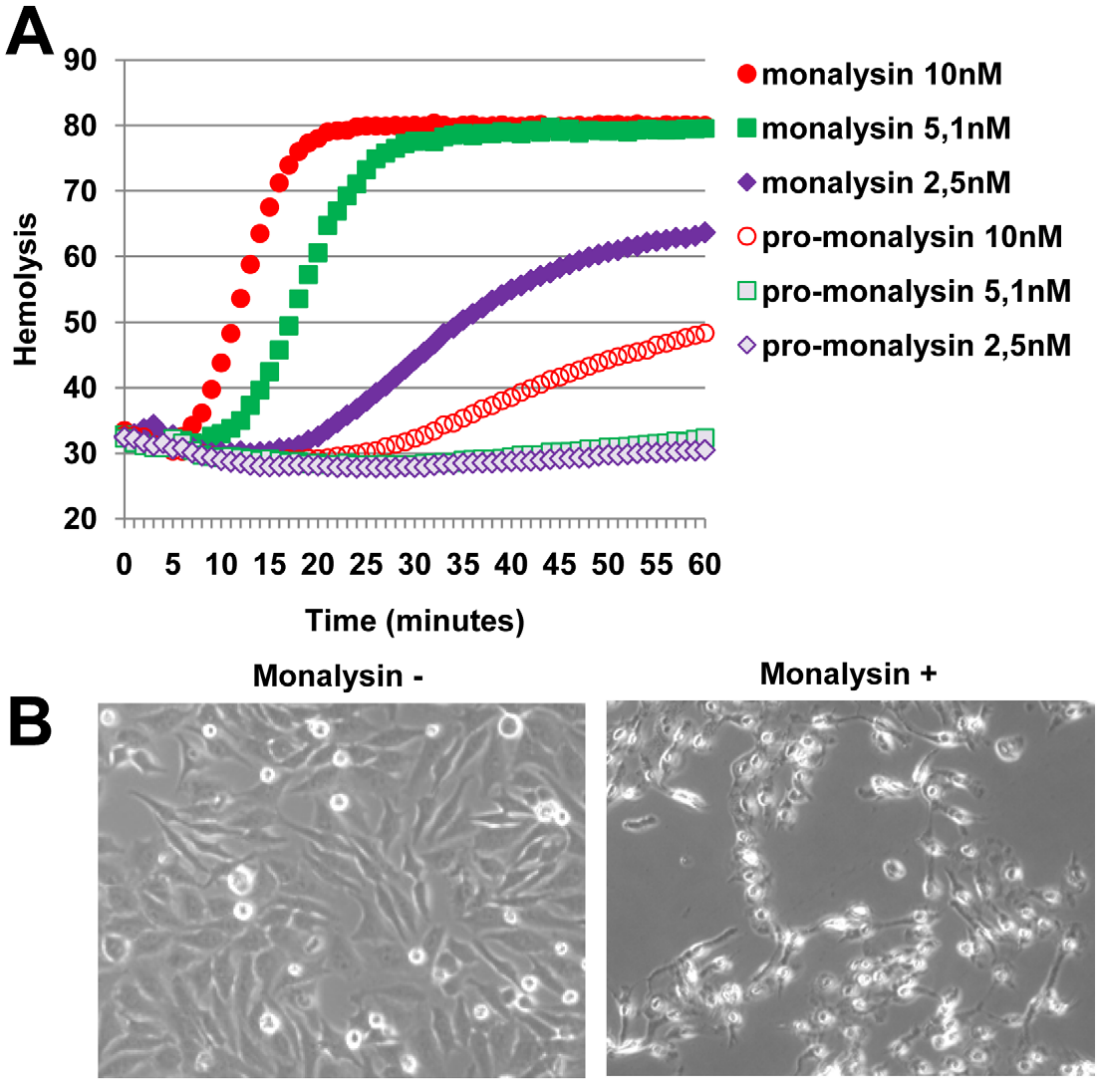
Monalysin hemolytic activity and cytotoxicity towards mammalian cells. (**A**) Hemolytic activity was measured with pro and matured Monalysin incubated with red blood cells. The mature Monalysin was obtained by limited trypsinolysis of a fresh extract of recombinant Pro-Monalysin. (**B**) Phase contrast microscopy of Hela cells untreated or treated with Monalysin 100 nM for 24h. Cells were shrinking and displayed irreversible loss of adherence.

In order to characterize the activity of the secreted protein encoded by *monalysin*, we produced and purified a his-tag version of it in *E. coli*. Ingestion of the recombinant protein at high dose had no impact on fly survival. However, the Monalysin protein was highly toxic when directly injected in the body cavity (**Figure 3A**). This dose-dependent lethal activity suggests that Monalysin function as a bacterial toxin. Results shown in **Figures 3B, 3C** **and Table S2** indicate that Monalysin has a strong cytotoxicity towards S2 cells (derived from *Drosophila* embryonic hemocytes) and SF9 cells (from the Lepidoptera *Spodoptera frugiperda*). Moreover, Monalysin treated S2 cells showed DNA fragmentation and condensation that are sign of apoptosis (**Figure 3D and E**). Along the same line, **Figure 4A** shows that the recombinant toxin rapidly induced hemolysis in a dose dependant manner as measured by the loss of sample turbidity. In addition we found that two mammalian culture cell lines – Hela and RPE1– were also sensitive to Monalysin (**Figure 4B** **and Table S2**). Altogether these observations show that Monalysin is a secreted cytotoxic factor of *P. entomophila* with a broad range of activity.

### Regulation and processing of Monalysin

**Figure 5:**
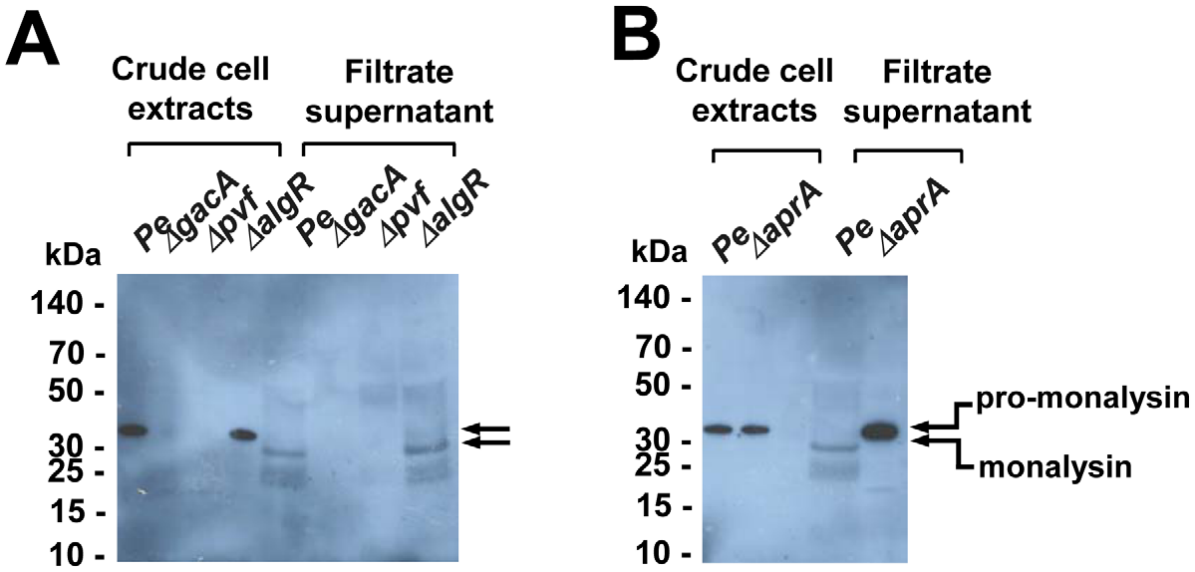
Regulation and processing of Monalysin. (**A**) Western-blot analysis of proteins from crude cell extracts or filtrate supernatants shows that Monalysin was not produced in *gacA* and *pvf* mutants, but was produced in the *algR* mutant. (**B**) Western-blot analysis of bacterial crude cell extracts and filtrated supernatants of *Pe wt* and *ΔaprA* shows that pro-monalysin is not processed in the supernatant of an *AprA* mutant. The stronger signal in the *AprA* mutant lane is due to the fact that the serum recognized better the pro-monalysin than the monalysin (see **Figure S5**).

*P. entomophila* virulence is controlled by several regulatory systems: *i)* the two component system GacS/GacA that functions at the post-transcriptional level, *ii)* a secreted signaling molecule produced by the *pvf* genes, and *iii)* AlgR that is known to control alginate production in other bacteria [5], [6], [7]. To determine which of these mechanisms regulate Monalysin production, crude cell extracts or filtered supernatants from wild-type and mutant *P. entomophila* were analyzed using a specific antiserum **(** **Figure 5A** **, Figure S5)**. Monalysin was undetectable in both cells and medium of *P. entomophila* lacking the two-component system GacS/GacA and the *pvf* signaling molecule.

**Figure 6:**
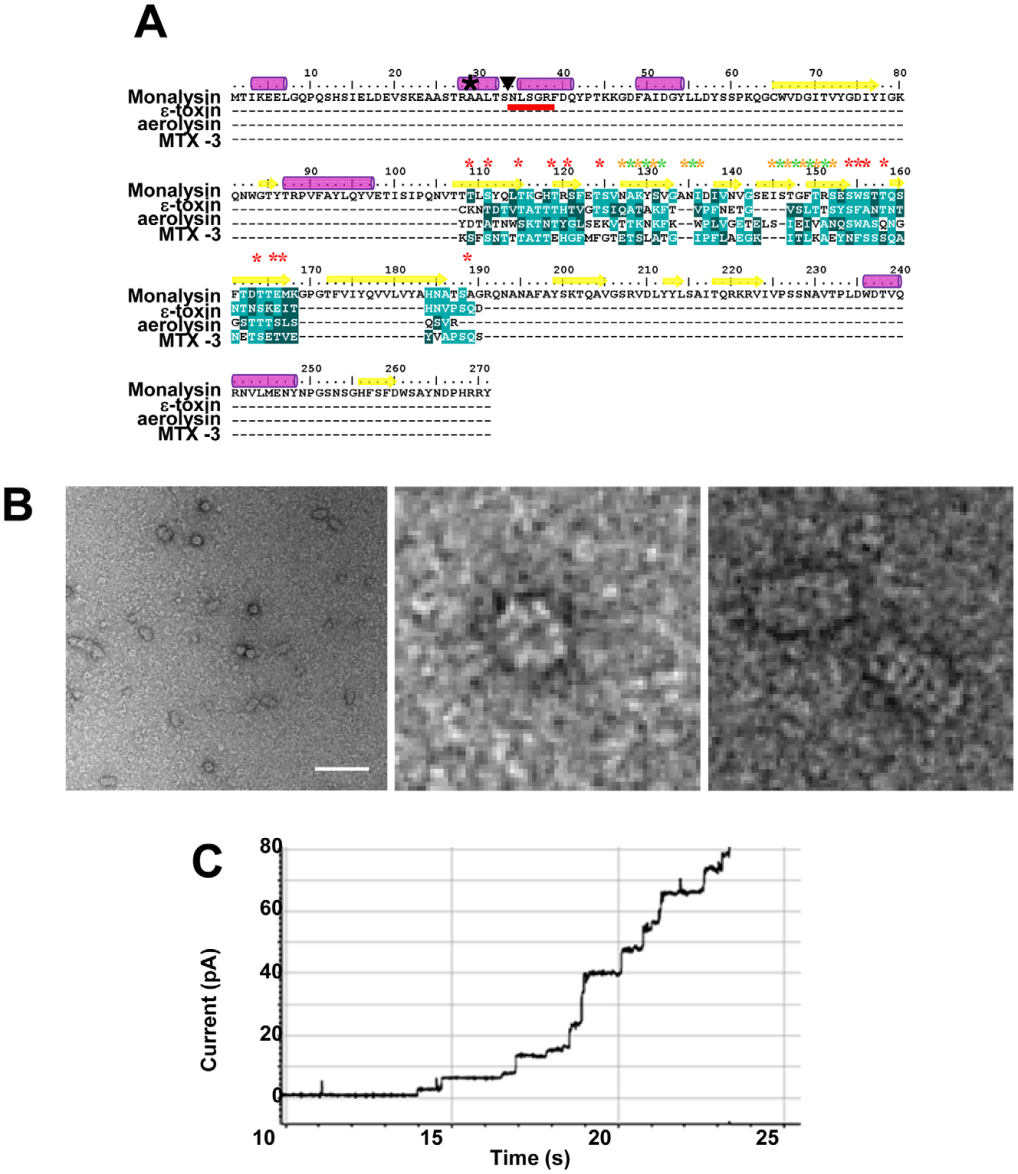
Monalysin is a *ß* pore-forming toxin. (**A**) Protein sequence analysis of Monalysin reveals an internal domain with amphipathic patches flanked by serine- and threonine-rich sequences that shares similarities with the membrane-spanning domain of ß-PFT (ε-toxin from *Clostridium perfringiens*, aerolysin from *Aeromonas hydrophila* and MTX-3 from *Bacillus sphaericus*). The multiple sequence alignment reveals the presence in *P. entomophila* Monalysin of putative membrane exposed residues (Yellow stars), solvent-exposed residues (green stars), and serine and threonine residues (red stars). A black star (★) shows the first amino acid detected by MALDI-TOF analysis of tryptic fragment of the recombinant Monalysin. The N-terminal residues of the mature Monalysin, identified by Edman sequencing, present in *P. entomophila* supernatant are underlined in red; a triangle (▾) indicates the potential cleavage site of pro-monalysin deduced from the N-terminal Edman sequencing. Purple cylinders indicate predicted α-helixes and yellow arrows indicate predicted β-sheets. (**B**) Scanning Electron micrographs show that Monalysin forms circular-like structures (top view) and barrel-like aggregates (side view). Scale bar represents 100 nm. (**C**) Monalysin (5 µg.ml) is able to form pores in a planar lipid bilayer.

Interestingly, this analysis revealed that the supernatant of *P. entomophila* contained a shorter form of Monalysin when compared to the form detected in cell extracts. A N-terminal Edman sequencing of the shorter form found in culture supernatant (extracted from SDS gels) was performed, which revealed that the size shift was due to a cleavage taking place before Asparagine 34 (indicated in **Figure 6A**). Many toxins require a proteolytic activation, which can be performed by proteases produced by the bacterium itself or by enzymes of the host digestive tract [17]. Interestingly *P. entomophila* secretes large amounts of the metallo-protease AprA. To test whether AprA could be responsible for maturation of pro-Monalysin to Monalysin, we analyzed the supernatants of *AprA*-deficient and wild-type *P. entomophila* by Western blotting. **Figure 5B** shows that pro-Monalysin was found in supernatant derived from the *aprA* mutant while the mature form predominates in supernatant from wild-type *P. entomophila*.

Collectively, our data indicate that Monalysin production is controlled by the global regulatory systems Gac and Pvf and that its N-terminus is cleaved by AprA upon secretion into the extracellular medium.

### Monalysin is a novel *ß*-type pore-forming toxin

The Monalysin amino acid sequence does not show any homology to other sequences using P Blast, except for two uncharacterized orthologs found in *Pseudomonas putida* F1 strain (**Figure S1**). Neither the *P. entomophila* nor the *P. putida* gene products displayed any obvious protein domains. Nevertheless, the use of the HHpred software (Homology detection & structure prediction by HMM-HMM comparison) revealed the presence of an internal region with alternating polar and hydrophobic residues flanked by stretch of serine- and threonine residues, a hallmark of the membrane-spanning region of *ß*-barrel pore-forming toxins (**Figure 6A**). PFTs can be classified according to the secondary structure of their membrane-spanning region as α- and ß-PFTs. Far-UV circular dichroism analysis of Monalysin revealed a spectrum typical of structured proteins (**Figure S2**). The content of α-helixes and β-sheets was estimated to be 13% and 40%, respectively in agreement with the secondary structure prediction obtained with the program JPRED giving 17% of α-helixes and 35% of β-sheets as indicated in **Figure 6A**. This program also indicated that the putative membrane-spanning region of Monalysin was formed of a ß-sheet. This sequence analysis suggests that Monalysin is related to PFT of the ß-type.

ß-PFTs are synthesized as soluble proteins and have the ability to multimerize into circular polymers at high concentration, a step that for certain toxins, such as Aerolysin, requires proteolytic activation [18]. We next investigated whether Monalysin shared these properties with PFTs. SDS-PAGE analysis of a fresh recombinant Monalysin solution revealed a major band at the expected size (30 kDa) as well as several high molecular weight bands corresponding to oligomers that were resistant to SDS (see below). Interestingly, a shorter form of the protein was observed upon storage of samples at 4°C **(Figure S3A)**. This together with the observation that Monalysin is matured by AprA indicates the existence of a protease sensitive site in the N-terminus part of Monalysin (**Figure** **5B**). The cleavage of the recombinant pro-Monalysin into its shorter form could also be induced by a limited trypsinolysis (**Figure S3B)**. This processed form has a molecular weight of 26.5 kDa as determined by MALDI-TOF analysis, as opposed to 30.2 kDa for the full-length pro-toxin. Interestingly, Monalysin had stronger hemolytic activity than pro-monalysin (**Figure 4A**), indicating that the removal of the N-terminal fragment constitutes a maturation step that enhances the cytotoxic activity. Processing of pro-monalysin to its mature form was accompanied by an increase and change in the higher order SDS-resistant complexes (**Figure S3B)**.

While multiple size oligomers were observed by SDS-PAGE, a single species was observed by native PAGE analysis of Monalysin (**Figure S3C**). Multi-Angle Light Scattering analysis (MALS/UV/RI) confirmed the presence of a single species with a molecular mass of 546 kDa and hydrodynamic radius of 7.5 nm hence a diameter of 15 nm, which would correspond to about 18 monomers (**Figure S4**). This was further confirmed by electron microscopy of negatively stained recombinant Monalysin which showed circular like (top view) and barrel like structures (side view) similar to that observed with other ß-PFT (**Figure 6B**).

Sequence analysis of Monalysin, its ability to form ring like high order structures combined with its hemolytic activity strongly indicate that the toxin is a PFT. To address this issue directly, we analyzed its ability to form channels in planar lipid bilayers, an extremely sensitive electrophysiological method that enables the study of single-channel events. Addition of Monalysin led to a stepwise increase in membrane current, reflecting the formation of pores (**Figure 6C**). Collectively, our data show that *P. entomophila* Monalysin is a *bona fide* pore-forming toxin of the ß type.

### **Discussion**

Many bacterial pathogens, both Gram-positive and Gram-negative, produce PFTs that contribute to their virulence [17]. Here we report the identification of a novel PFT that contributes to the virulence of *P. entomophila* against *Drosophila*. Our data show that Monalysin requires N-terminal cleavage to become fully active, forms oligomers *in vitro*, and induces pore formation in artificial lipid membranes. The prediction of the secondary structure of the membrane-spanning domain indicates that Monalysin is a PFT of the ß-type. Outside of this domain, Monalysin does not show any homology to any other PFT and appears rather different from previously identified insecticidal PFTs such as *B. thuringiensis* Cry toxins. Nevertheless, Monalysin has two homologs in the closely related *P. putida* F1 strain. These proteins could participate to the interaction of some *Pseudomonas* species with eukaryotic cells, defining a new family of PFTs.

We previously showed that *P. entomophila* virulence is multi-factorial and regulated by multiple signaling modules. Taking advantage of the genetic amenability of both the host and the pathogen, we aimed to identify *P. entomophila* and *Drosophila* pathways and effectors involved in the infectious process. Using this integrated approach, we previously proposed a role for the AprA metalloprotease in protection against antimicrobial peptides [7]. We now identify a second virulence factor, the ß-PFT Monalysin. Like AprA, a *mnl* mutant is affected in several, but not all, aspects of *P. entomophila* virulence. This attenuated virulence of the *mnl* mutant is clearly shown by survival analysis, which monitors the global outcome of infection. Our study indicates that Monalysin significantly contributes to the damage inflicted to intestinal cells by the bacterium, which is fully consistent with its activity as a pore-forming toxin. Supporting this notion, we observe that a *mnl-*deficient mutant induced less cell damage and a lower level of stress and repair pathway activity. The *mnl* mutant still induced a local immune response but the systemic immune response is drastically attenuated. This is also consistent with a role of Monalysin as a cytotoxin since activation of a systemic immune response is probably linked to damage of intestinal tract rendering possible the translocation of peptidoglycan, the bacteria elicitor activating the Imd pathway, from the lumen to the hemolymph compartment.

We also show that Monalysin production is regulated both by the GacS/GacA two component-system and the *pvf* genes. However, a *mnl* mutant still causes higher levels of stress and damage to the intestinal epithelium than *gacA* or *pvf* mutants. This indicates that these signaling modules regulate additional virulence factors contributing to *P. entomophila* cytotoxicity. Alternatively, it is possible that the overall cytotoxicity is caused by a synergy between the metalloprotease AprA and the PFT Monalysin, both of them being regulated by GacA. Along this line, we observed that AprA promotes the rapid cleavage of the pro-Monalysin into its active form. Since Monalysin can also be processed by trypsin, it is likely that AprA is not essential for PFT function of Monalysin in the *Drosophila* gut, as this toxin could also be processed by host enzymes. Both Monalysin and AprA are expressed in the *algR* mutant that affects a transcriptional regulator involved in alginate production as well as genes that are often associated to virulence (*ie*: pili biosynthesis, cyanide production…) [19]. The observation that an *algR* mutant is still avirulent (Vodovar 2005), although expressing both Monalysin and AprA, indicates the existence of additional virulence factors. Future studies should investigate at which level Pvf and GacS/GacA affect Monalysin production as well as identify other potential virulence factors regulated by the Pvf, Gac or AlgR.

Recent studies have shown that cells respond to PFTs by inducing repair and stress signal-transduction pathways to repair damage. Studies in *C. elegans* and mammalian cells have revealed a role for the P38 pathway, the unfolded protein response, and hypoxia in cellular resistance to the action of PFT [20]–[22]. The reduced expression of JAK-STAT and JNK pathway activities in guts infected with the *mnl* mutant indicate that *Drosophila* epithelial cells respond to PFT by activating stress and repair pathways. Thus, the *P. entomophila/Drosophila* interaction provides an interesting model to dissect the host response to PFTs in a natural infectious context.

Insects are potential reservoirs for microbes and are ideal vectors for their transmission due to their motility and their capacity to live in bacteria-rich environments [23]. This is exemplified by fruit flies that live in rotting fruits and are capable of transmitting phytopathogenic bacteria [24]. Insects are notably resistant to microbial infection allowing them to colonize these microbe-rich environments. This is largely due to the existence of very efficient physical barriers that block entry of microbes in the body cavity. As an illustration, injection of less than 10 cells of *P. aeruginosa* or *Serratia marcescens* in the body cavity rapidly kills flies, while high doses of these bacteria have only modest effects on survival when ingested [25]. In contrast to mammals, the gut of insects is lined with a chitinous matrix, the peritrophic matrix [26], that blocks the direct interaction between bacteria and epithelia cells and prevents the use of virulence devices such as type III and VI secretion systems that allow the injection of virulence factors directly into target cells. Rare bacterial species such as *Photorhabdus luminescens* can bypass this physical barrier since there are transported by symbiotic nematodes that can pierce the insect cuticle [27]. Other entomopathogens that enter through the oral route have to escape the local immune response and breach the gut barrier [23]. Despite the characterization of several virulence factors in few species, the mechanisms by which enteric pathogens kill insects remain poorly understood. This paper together with the well-characterized action of *Bacillus thuringiensis* cytotoxin Cry suggests that PFTs efficiently promote bacterial colonization of the insect gut [28]–[30]. This heavy artillery strategy does not require a direct contact between bacteria and host cells since PFTs can cross the pores of the peritrophic matrix and reach intestinal cells. PFTs can induce gut damage and rupture of intestinal homeostasis that *in fine* will lead to a weakening of the gut barrier and an inhibition of gut peristaltism promoting bacteria persistence. Gut damage and food uptake blockage are two symptoms of insect pathogenesis and could reflect the action of PFTs [23]. It would be interesting to know if other entomopathogens such as *Serratia marcescens* and *Serratia entomophila* also used PFT to colonize their insect host. In conclusion, this and other studies using different bacteria species contribute to uncovering strategies used by entomopathogens to breach insect barriers. A better knowledge of these strategies could also open the route to new methods of insect pests control.

## Materials and Methods

### Bacterial strains, media and antibiotics

*P. entomophila* L48 [4] was grown in LB for all experiments. *P. entomophila* mutated for the *gacA*, *aprA*, *algR*, and the *pvf* gene are described elsewhere [4], [5], [6], [7], [11]. The *mnl* deletion construct was generated by amplifying flanking regions of the *monalysin* gene (*pseen3174* or *mnl*) by PCR. The resulting PCR product was cloned into the plasmid pEXG2. This plasmid was then used to create the strain *Δ3174* (alternatively *Δmnl)*, containing a deletion of the gene *pseen3174*. Complementation construct were made by cloning into the plasmid pPSV35 of PCR-amplified DNA fragments from *P*. *entomophila* containing the mutated genes. *Pseudomonas* Isolation agar (PIA, Difco) was used for selection after conjugations and persistence experiments. When *E. coli* was grown, antibiotics were used when necessary at the following concentrations: G418, 25 µg/ml and tetracycline, 5 µg/ml. When *P. entomophila* was grown, antibiotics were used when necessary at the following concentrations: gentamicin, 50 µg/ml for liquid cultures and 150 µg/ml for solid media, tetracycline 40 µg/ml and rifampicin, 30 µg/ml. The bacterial strains used in this study and the culture conditions are presented in **Table S1**. All primer sequences are available upon request. Insertion constructs were generated as previously described [6], [11].

### Sequence analysis

DNA sequence searches and analysis were performed using the *Pseudomonas* genome database (www.pseudomonas.com). The *monalysin* gene (ORF PSEEN3174) corresponds to the accession number YP_608728.1 . Monalysin putative orthologs in *Pseudomonas putida* Pput_1063 and Pput_1064 correspond to the accessions numbers YP_001266408.1 , YP_001266409.1 respectively. The ORF PSEEN0535 involved in the production of the type VI secretion system corresponds to the accession number YP_606298.1 Monalysin amino-acids sequence analysis was performed using the HHpred software (Homology detection & structure prediction by HMM-HMM comparison http://toolkit.tuebingen.mpg.de/hhpred).

### Fly stocks and infection assays

Oregon R flies were used as a standard wild-type strain and were maintained at 25°C. Adherens junctions were visualized using *ubi-DE-cadherin-GFP* flies [14], [31]. *Upd3* expression in unchallenged gut and following infection, was monitored using *upd3-Gal4, UAS-GFP* flies (Buchon et al., 2009). Fly natural infections were carried out at 29°C on 4- to 8- day-old adult females as previously described. All the infections, except when specified, were carried out with bacterial preparation adjusted to an OD600 = 100 which correspond to 6.5E10 colony forming units per ml [11]. Monalysin was injected in the body cavity of fly using a Nanodrop microinjector (Nanoject). Virulence assays were performed at least three times in triplicate.

### Reverse transcriptase quantitative PCR analysis

Total RNA was extracted from whole flies (5 for each assay) or from dissected guts without Malpighian tubules (14 for each assay) using TRIzol (Invitrogen). RT-qPCR was performed using SYBR Green I (Roche) on a Lightcycler 2.0 (Roche) as previously described [32]. Data represent ratio of the amount of mRNA detected normalized to the amount of the control *rpl32* mRNA. Experiments were performed at least three times independently. Averages of more than three experiments are shown.

### Cell cultures, treatments, cytotoxicity assays and live imaging

The macrophage-like lineage S2 cells derived from *D. melanogaster* embryos where grown in Schneider's medium (Invitrogen). The Sf9 cells (Invitrogen) derived from *Spodoptera frugiperda* (Lepidoptera) pupal ovarian tissue were cultured in complete TNM-FH (Invitrogen). The mammalian cell lines Hela and the Retinal Pigmented Epithelial (RPE1) were grown in a humidified incubator with 5% CO_2_ at 37°C. Hela cells were cultured in MEM media supplemented with 10% fetal calf serum, 1% penicillin-streptomycin, 1% glutamine and 1% NEAA (Gibco). RPE1 cells were cultured in DMEM media supplemented with 10% fetal calf serum, 1% penicillin-streptomycin and 1% glutamine (Gibco). Cell viability was observed using the LIVE/DEAD Viability/Cytotoxicity Assay Kit (Invitrogen) according to the provider instruction. Briefly cells are simultaneously labeled with calcein AM that reveals intracellular esterase activity in live cells and ethidium homodimer (EthD-1) that reveals plasma membrane damages. LDH release from damaged cells was measured following the instructions of the CytoTox-One Homogeneous Membrane integrity Assay kit (Promega)****. *In Situ* Nick Translation was performed to detect fragmented DNA in nuclei. *In situ* DNA synthesis was performed by a DNA polymerase I (150 units/ml) (Takara) in the presence of a dNTP mix in which dUTP is tetramethylrhodamine-conjugated (Roche). The reaction was carried out for 90 min at room temperature. Live imaging and immunofluorescence were performed as previously described [8]. After treatment, cells were recovered, fixed with 4% PFA and permeabilized with 0.3% Triton X-100. Dead cells were detected using acridine orange staining (Invitrogen). Dead cells quantification was performed as follows: 16 hours after infection, guts were dissected and stained with acridine orange and DAPI. Pictures were taken using a fluorescent microscope. From these pictures, groups of 100 hundred DAPI stained nuclei were randomly defined and the number of acridine orange positive nuclei (*ie* dead cells) was determined. Three parcels per guts were analyzed. The results are the mean of four independent experiments. Nuclei were stained by DAPI (Sigma). All the images were performed using a Zeiss Axioimager Z1.

### Monalysin expression, purification, and analysis

All cloning steps were performed as described earlier [33]. The sequence of *Monalysin* (from residue 1 to 271, access number *pseen3174*) was PCR-amplified from genomic DNA (isolated from *P. entomophila)* and cloned into pDONR201 (Invitrogen). The ORF was then subcloned into the pETG-20A *E. coli* (a generous gift from Dr A. Geerlof, EMBL) destination vector to generate a constructs encoding Monalysin with an N-terminal fusion composed of the thioredoxin (TRX) protein, followed by a 6xHis-tag and a Tobacco Etch Virus (TEV) protease cleavage site. The construct was sequenced verified. The production and purification were performed as described earlier [34]. Briefly, the pETG-20A-*Monalysin* was transformed into Rosetta (DE3) pLysS *E. coli* cells (Novagen). An overnight LB pre-culture (with 100 mg mL-1 ampicillin and 34 mg mL-1 chloramphenicol) was used to inoculate large cultures in ZYP-5052 auto-induction media [35] supplemented with the same antibiotics and incubated with vigorous shaking (250 rpm) at 37°C during 4 h. At this stage, the temperature was decreased to 17°C, and the cultures were allowed to grow for an additional 18 h with vigorous shaking (250 rpm). After 18 h, cells were harvested by centrifugation (4000*g* for 10 min) and the pellet was homogenized and frozen in lysis buffer (50 mM Tris-HCl; 500 mM NaCl; 0.5 mM lysozyme; 10 mM imidazole and 1 mM phenylmethylsulfonyl fluoride (PMSF), pH 8). The cell pellets were thawed and lysed with a sonicator after the addition of DNase I at 20 mg mL^−1^ and 1 mM MgSO_4_. The pellet and soluble fraction were separated by centrifugation (30 min at 16,000*g*) of an early stationary phase culture. The supernatants were filtered through a 0.22 µm filter and were concentrated 50-fold by using 5 kDa cutoff Centricon membranes (Biorad). The cell pellets were washed in PBS, resuspended in PBS containing protease inhibitors and lysed by sonication. The soluble fraction was purified by immobilized metal ion affinity chromatography using a 5 mL HisTrap crude (GE Healthcare) Ni^2+^-chelating column equilibrated in buffer A (500 mM NaCl; 50 mM Tris–HCl; 10 mM imidazole; pH 8). After the loading of the soluble fraction and a column wash (buffer A with 50 mM Imidazole), the protein was eluted with buffer A supplemented with 250 mM imidazole. The eluted fraction was desalted in buffer A (Hiprep 25/10 Desalting column, GE) and the protein concentration of the TRX-His6-TEV-Monalysin determined. After a 4°C overnight cleavage of the protein with 1∶20 w:w His-TEV protease, the TRX-His6-TEV and the His-TEV were separated from the pure Pro-Monalysin by collecting the Flow Through (FT) of a second Nickel purification. The final purification and the characterization of the oligomeric state of the monalysin were achieved by the separation of the FT on a size exclusion chromatography (HiLoad 16/60 Superdex 200 prep grade, GE), equilibrated in Tris 10mM, NaCl 500mM pH8. The pure Pro-Monalysin was used for the functional characterizations. For the MultiAngle Light Scattering analysis, size exclusion chromatography was carried out on an Alliance 2695 HPLC system (Waters) using a Silica Gel KW804 column (Shodex) equilibrated in 10 mM Tris and 150 mM NaCl at pH 7.5 at a flow of 0.5 ml/min. Detection was performed using a triple-angle light scattering detector (Mini-DAWN TREOS, Wyatt Technology), a quasi-elastic light scattering instrument (Dynapro, Wyatt Technology), and a differential refractometer (OptilabrEX, Wyatt Technology). Proteins were analyzed by SDS-PAGE. Native PAGE was performed to determine the oligomeric state of Monalysin. To generate Monalysin, pro-monalysin samples were submitted to limited trypsinolysis by adding trypsin (1∶100 w:w). The reaction was stopped by using a trypsin-chymotrypsin inhibitor (Invitrogen). Pro-monalysin and Monalysin were detected by Western-blot using a specific serum that recognized better the pro-monalysin than the Monalysin **(see Figure S5)**.

### Production of the antibody anti-monalysin

The Guinea pig antibody anti-Monalysin was provided by Eurogentec.

### Circular dichroism, molecular weight and hydrodynamic radius determination

Far-UV Circular Dichroism (CD) spectra (**Figure** **S2**) were recorded with a JASCO J-810 spectropolarimeter (JASCO Corporation) equipped with a Peltier temperature control and using 1-mm thick quartz cells. The molecular weight of recombinant pro-monalysin and Monalysin was determined by MALDI-TOF/TOF. Molecular weight and hydrodynamic radius determination was performed by the ASTRA V software (Wyatt Technology). Proteins were loaded at a final concentration of 0.02 mM.

### Edman sequencing

After SDS-PAGE electrophoresis and Coomassie blue staining, protein bands were excised. Proteins were extracted from gel and blotted onto polyvinylidene difluoride membranes with the ProSob system (Applied Biosystems). The N-terminal sequences of proteins were determined by automated Edman degradation by introducing the blots into a Procise P494 automated protein sequencer (Applied Biosystems). The sequences obtained were compared to sequences in public protein sequence databases.

### Planar lipid bilayer

Planar lipid bilayer experiments were performed as previously described [36]. The bilayer was formed by painting a solution of 50% PC (egg lecithin) / 50% DOPE (w:w) in n -decane (40 mg ml ^−1^) on an aperture (d = 150 µm, pretreated with the same solution) in a delrin cuvette separating two chambers, each containing 1 ml of 1 M NaCl, 5 mM CaCl_2_ 10 mM HEPES, pH 7 and agar bridge connection (1 M KCl) to Ag/AgCl electrodes (Warner Instrument Corp. Hamden, CT). Monalysin was added to the *cis* chamber at room temperature.

### Statistical analysis

Survival assays have been performed at least three times in triplicate. The Kaplan-Meier log rank test was used to determine statistical significance. Dashed brackets represent the significance between the different infections (*: p<0.05, **: p<0.01, ***: p<0.001, ns  =  not significant). RT-qPCR analysis and cell death quantification using acridine orange staining are averages of at least 4 independent experiments. Error bars indicate standard errors. Statistical analysis was performed using a Wilcoxon test, and letters indicate significantly different values (*P*<0.05).

## Supporting Information

Figure S1 **Identification of two putative Monalysin orthologs in** ***Pseudomonas putida F1*** **.** Alignment of Monalysin amino-acids sequence and the sequence of its putative orthologs in *Pseudomonas putida* F1 encoded by the ORF Pput_1063 and Pput_1064.
(TIF)

Figure S2 **Far-UV CD spectra of Monalysin.** The far-UV CD spectra were recorded with a JASCO J-810 spectropolarimeter (JASCO Corporation) equipped with a Peltier temperature control and using 1 mm thick quartz cells. CD spectra were averaged on three accumulations using a scanning speed of 50 nm/min. Measurements were performed between 190 and 260 nm at 20°C in 10 mM Hepes buffer pH 7.5, NaCl 150mM with a protein concentration of 1 mg/ml. Circular dichroism of Monalysin reveals a spectrum of a protein with alpha-helix and beta-sheets.
(TIF)

Figure S3 **Recombinant Monalysin is processed by a proteolytic cleavage. (A)** SDS-PAGE analysis of recombinant Monalysin. Lane 1: fresh sample, line 2: old sample. **(B)** Silver staining of a SDS-PAGE fresh monalysin samples untreated (lane 1) or treated with trypsin (v:v) 1∶10 (lane 2), 1∶100 (lane 3), 1∶1000 (lane 4). **(C)** Native gel electrophoresis shows that Monalysin migrates at a high molecular weight.
(TIF)

Figure S4 **Absolute molecular weight determination of the Monalysin oligomer by Static Light Scattering analysis.** The molar mass (left axis, dotted lines) and the UV_280nm_ absorbance (right axis, solid lines) are plotted as a function of the column elution volume. Monalysin measured mass and hydrodynamic radius are 546.5 KDa and 7.52 nm, respectively.
(TIF)

Figure S5 **Western blot analysis of Monalysin. (A)** Western-blot analysis of proteins from crude cell extracts and filtrate supernatant. *Pe*  =  *P. entomophila* wild-type strain, *Δmnl*  =  the monalysin deficient strain, *Δmnl-pPSVmnl  = * the *monalysin*-deficient strain carrying a plasmid expressing a wild-type copy of the *monalysin* gene, *Δmnl-pPSV  = * the *monalysin*-deficient carrying the plasmid pPSV35 without any insert. **(B and C)** Comparison of the serum anti-monalysin recognition of pro-monalysin and monalysin. **(A)** Coomassie staining and **(B)** Western-blot of the same samples. Pro-Monalysin was purified from *E. coli* a described in *Material and Methods* and used to immunize Guinea pigs. The serum recovered from the final animal bleed was tested on a fresh toxin purification containing mainly 2 and 8 µg of the pro-Monalysin (line 1 and 3 respectively) and a sample containing the same amounts of predominantly the mature form Monalysin (line 2 and 4) as shown by coomassie staining. The western blot shows that the serum recognizes more epitopes in pro-monalysin than in monalysin. Indeed, monalysin (**Figure S5C**, lane 4) could be detected only when the exposure time was increased, which resulted in a saturating signal for pro-monalysin.
(TIF)

Figure S6 **Survival analysis of wild-type Oregon flies following oral infection with various concentrations of bacteria.** Survival curves of flies infected with various concentrations of **(A)** the *P. entomophila* wild-type strain (*Pe*), **(B)** the *mnl* deficient strain (*Δmnl*), **(C)** the *gacA*-deficient strain (*ΔgacA*). UN  =  unchallenged. The number next to the bacterial strains indicates the concentration (Optical Density measured at 600nm) of the bacterial sample use for the infection. Survival assays have been performed at least three times in triplicate.
(TIF)

Figure S7 **Cell death in guts of infected flies monitored by acridine orange staining.** Guts were dissected from unchallenged female Oregon flies **(A)** or infected for 16 h with wild-type *P. entomophila* **(B)**, a *mnl* mutant **(C)** or a *gacA* mutant **(D)**, and stained with acridine orange. Scale bars represent 50 µm.
(TIF)

Table S1 **Bacterial strains used in this study.**
(TIF)

Table S2 **Monalysin cytotoxicity towards insect and mammalian cells.** Different culture cell lines were treated with the indicated concentration of Monalysin. Sensitivity (+) or resistance (−) to Monalysin was determined by phase contrast microscopy observation performed at 4 and 24 h. For HRBC (human red blood cells) the sensitivity was monitored by hemolytic activity as described in *Material and Methods*.
(TIF)
