# Supplementary figures and images for: Monalysin, a Novel ß-Pore-Forming Toxin from the Drosophila Pathogen Pseudomonas entomophila, Contributes to Host Intestinal Damage and Lethality
Source: PLoS Pathog. 2011 Sep 29;7(9):e1002259. doi: 10.1371/journal.ppat.1002259 (PMC3182943; doi:10.1371/journal.ppat.1002259)

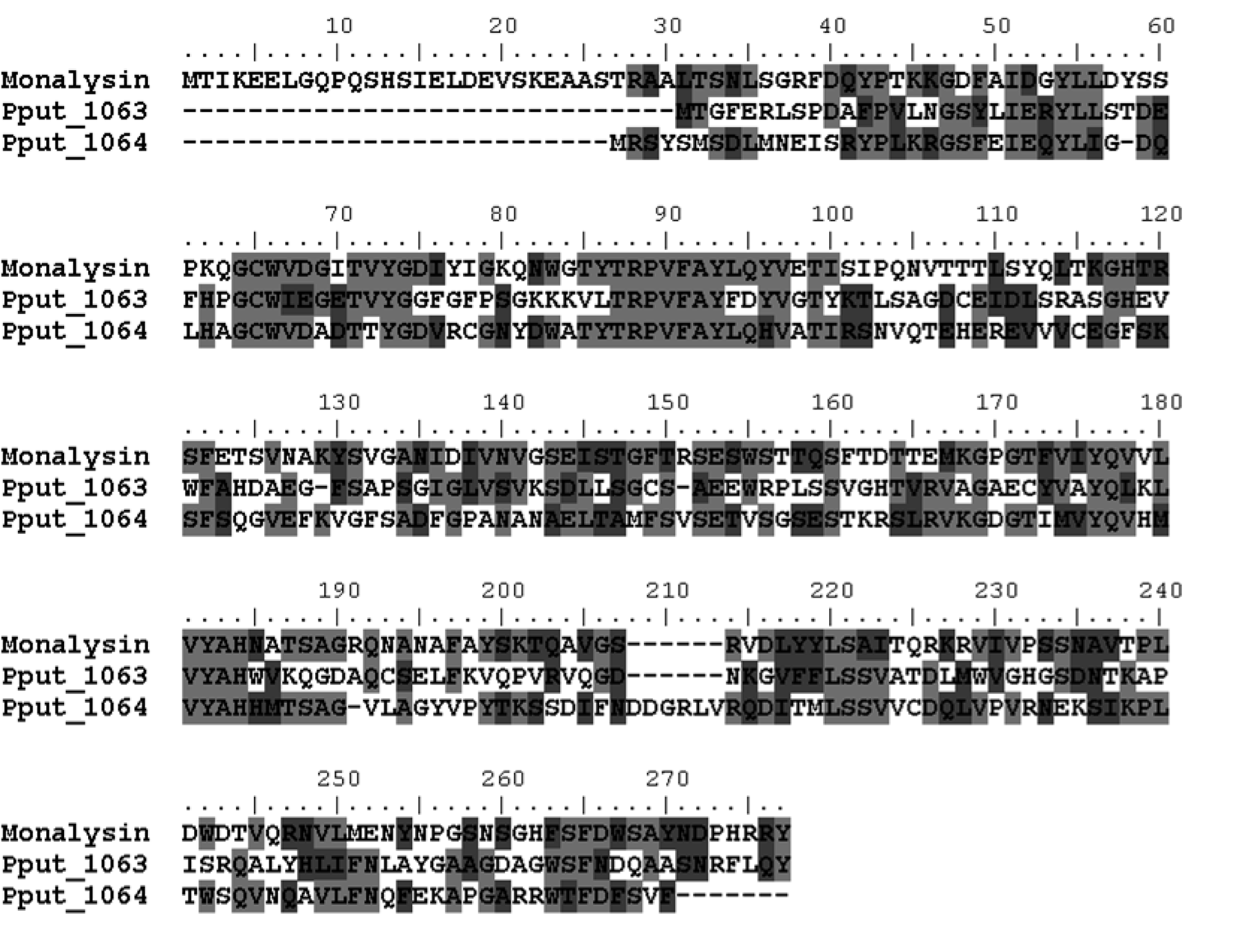

Supplement: Figure S1 — Identification of two putative Monalysin orthologs in Pseudomonas putida F1 . Alignment of Monalysin amino-acids sequence and the sequence of its putative orthologs in Pseudomonas putida F1 encoded by the ORF Pput_1063 and Pput_1064. (TIF) [file ppat.1002259.s001.tif]

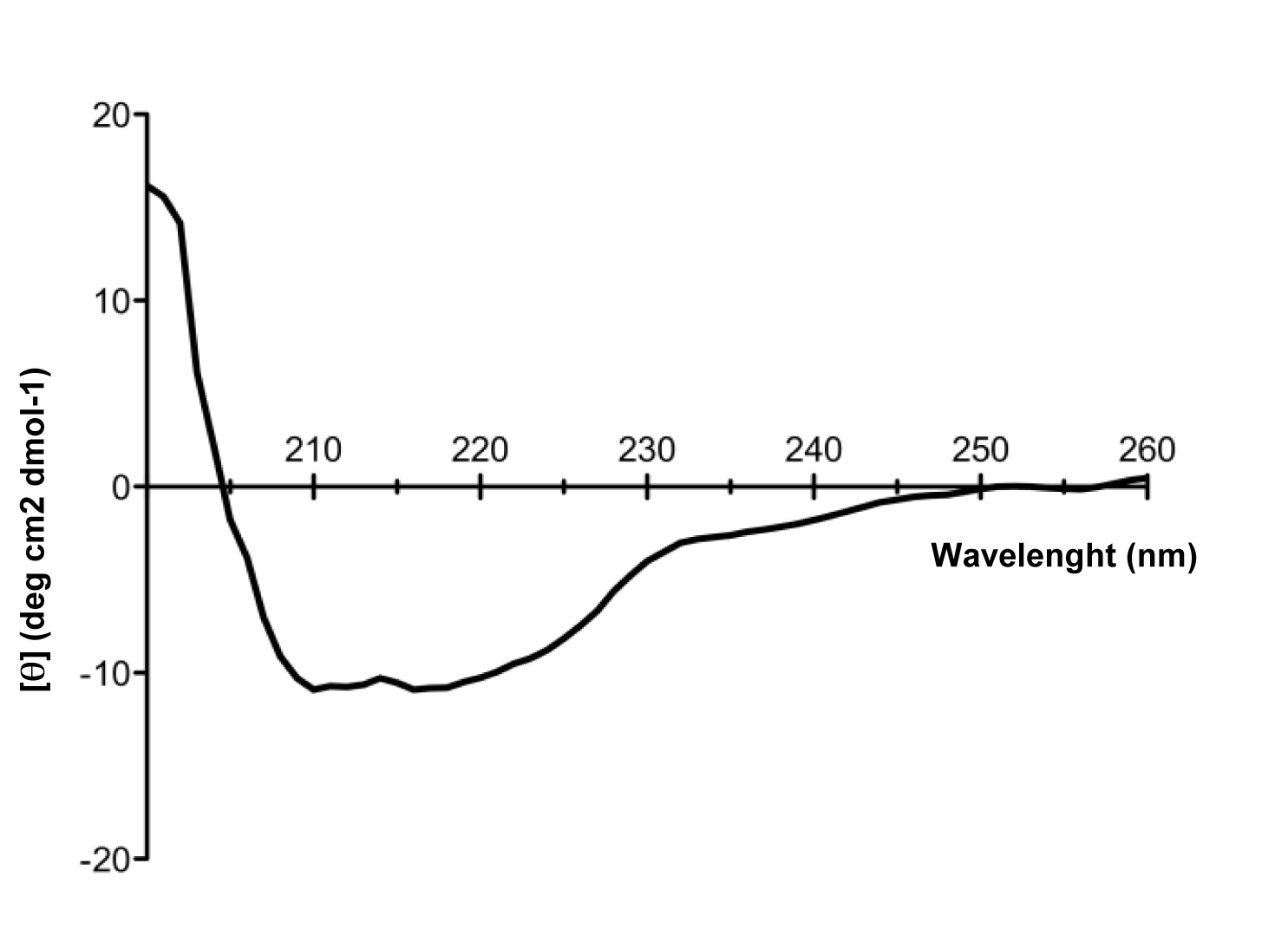

Supplement: Figure S2 — Far-UV CD spectra of Monalysin. The far-UV CD spectra were recorded with a JASCO J-810 spectropolarimeter (JASCO Corporation) equipped with a Peltier temperature control and using 1 mm thick quartz cells. CD spectra were averaged on three accumulations using a scanning speed of 50 nm/min. Measurements were performed between 190 and 260 nm at 20°C in 10 mM Hepes buffer pH 7.5, NaCl 150mM with a protein concentration of 1 mg/ml. Circular dichroism of Monalysin reveals a spectrum of a protein with alpha-helix and beta-sheets. (TIF) [file ppat.1002259.s002.tif]

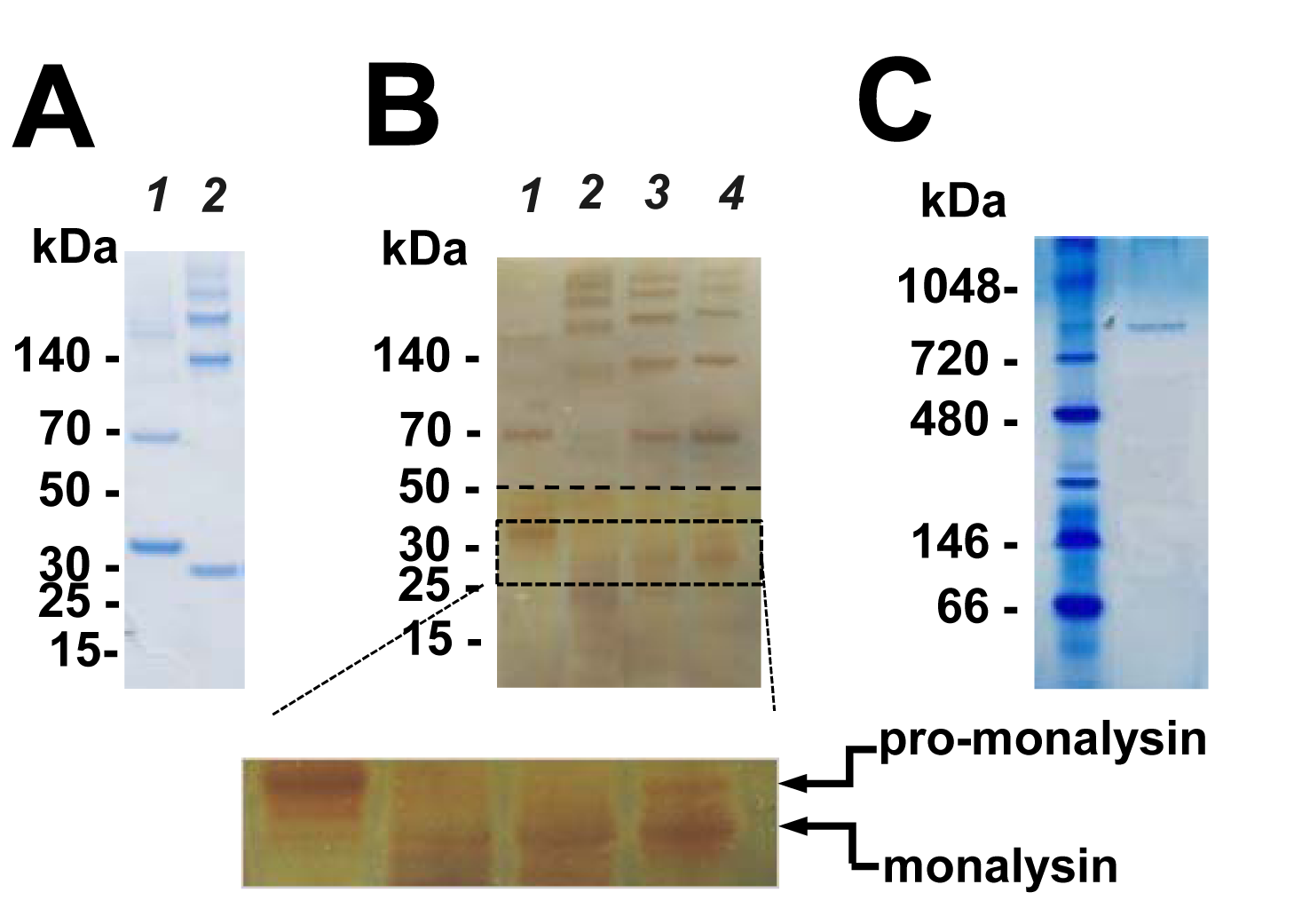

Supplement: Figure S3 — Recombinant Monalysin is processed by a proteolytic cleavage. (A) SDS-PAGE analysis of recombinant Monalysin. Lane 1: fresh sample, line 2: old sample. (B) Silver staining of a SDS-PAGE fresh monalysin samples untreated (lane 1) or treated with trypsin (v:v) 1∶10 (lane 2), 1∶100 (lane 3), 1∶1000 (lane 4). (C) Native gel electrophoresis shows that Monalysin migrates at a high molecular weight. (TIF) [file ppat.1002259.s003.tif]

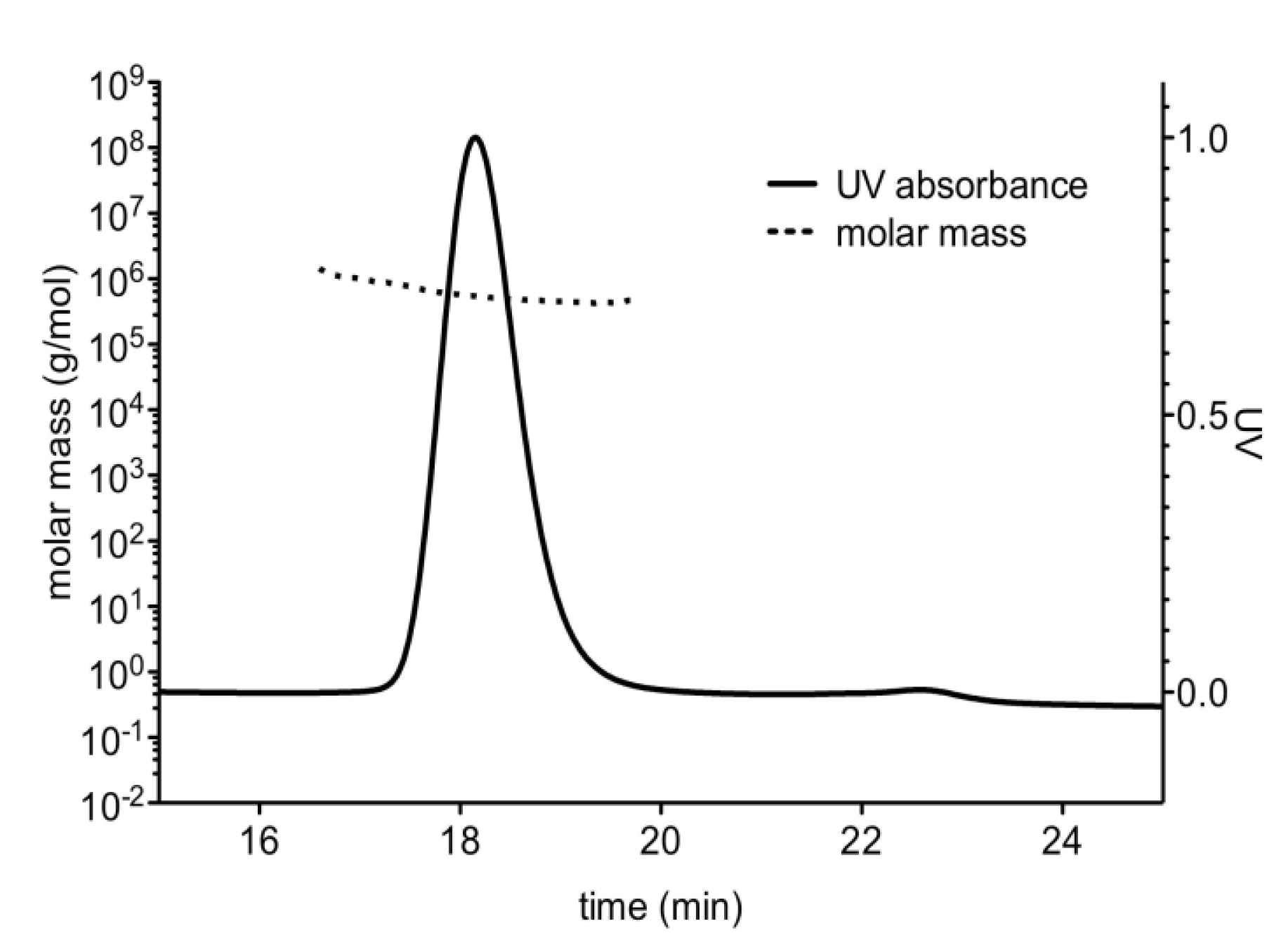

Supplement: Figure S4 — Absolute molecular weight determination of the Monalysin oligomer by Static Light Scattering analysis. The molar mass (left axis, dotted lines) and the UV280nm absorbance (right axis, solid lines) are plotted as a function of the column elution volume. Monalysin measured mass and hydrodynamic radius are 546.5 KDa and 7.52 nm, respectively. (TIF) [file ppat.1002259.s004.tif]

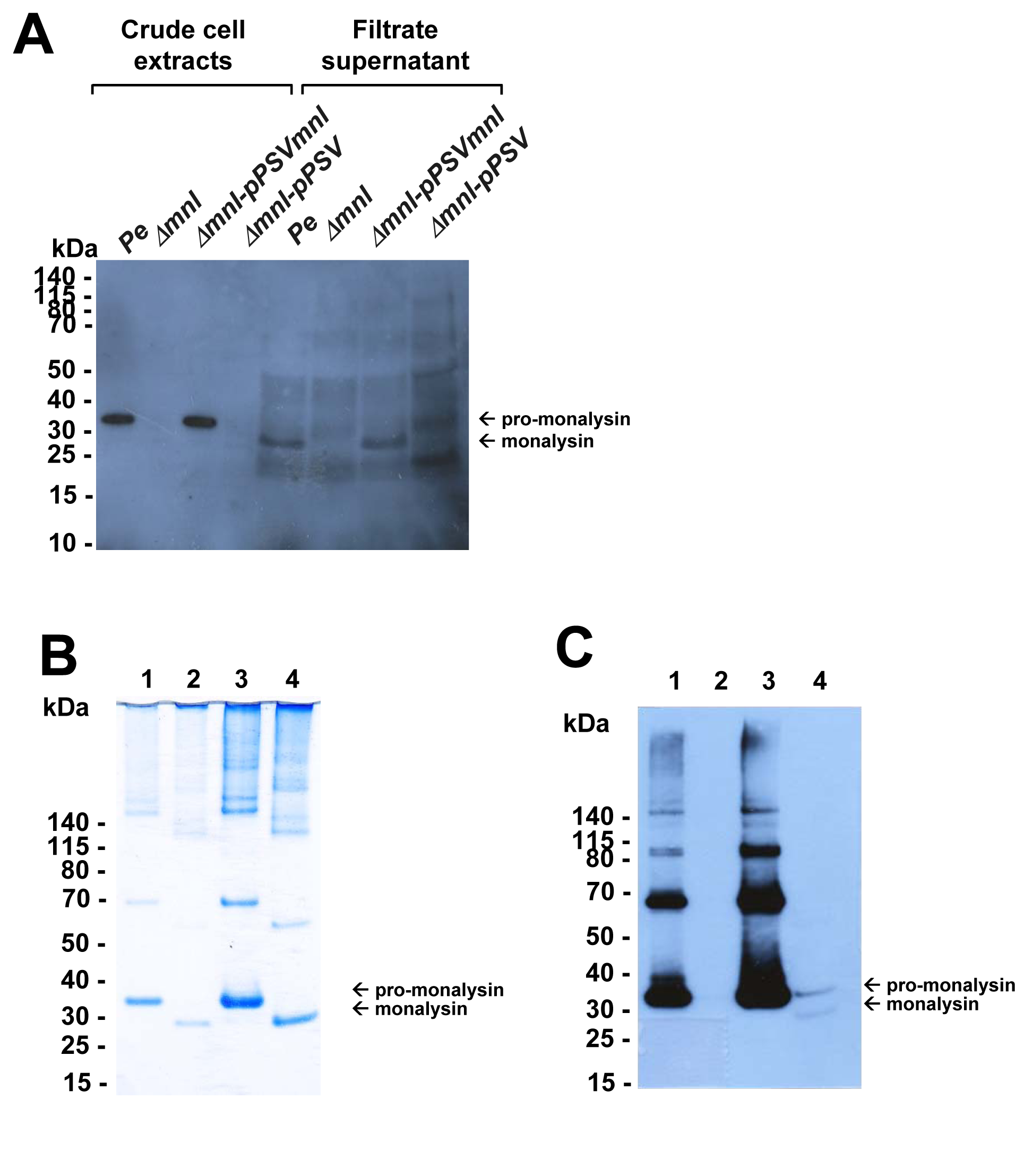

Supplement: Figure S5 — Western blot analysis of Monalysin. (A) Western-blot analysis of proteins from crude cell extracts and filtrate supernatant. Pe = P. entomophila wild-type strain, Δmnl = the monalysin deficient strain, Δmnl-pPSVmnl = the monalysin-deficient strain carrying a plasmid expressing a wild-type copy of the monalysin gene, Δmnl-pPSV = the monalysin-deficient carrying the plasmid pPSV35 without any insert. (B and C) Comparison of the serum anti-monalysin recognition of pro-monalysin and monalysin. (A) Coomassie staining and (B) Western-blot of the same samples. Pro-Monalysin was purified from E. coli a described in Material and Methods and used to immunize Guinea pigs. The serum recovered from the final animal bleed was tested on a fresh toxin purification containing mainly 2 and 8 µg of the pro-Monalysin (line 1 and 3 respectively) and a sample containing the same amounts of predominantly the mature form Monalysin (line 2 and 4) as shown by coomassie staining. The western blot shows that the serum recognizes more epitopes in pro-monalysin than in monalysin. Indeed, monalysin (Figure S5C, lane 4) could be detected only when the exposure time was increased, which resulted in a saturating signal for pro-monalysin. (TIF) [file ppat.1002259.s005.tif]

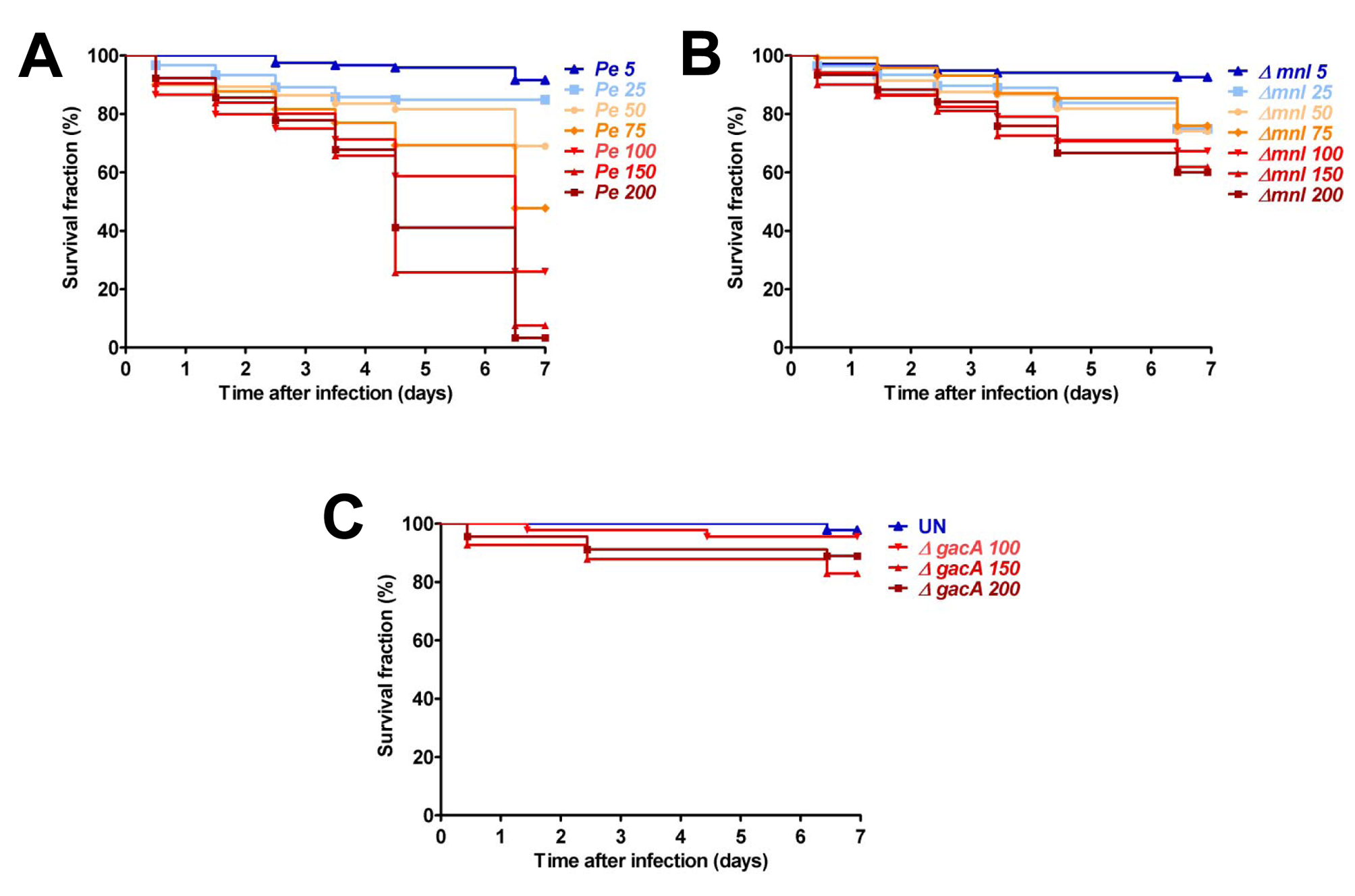

Supplement: Figure S6 — Survival analysis of wild-type Oregon flies following oral infection with various concentrations of bacteria. Survival curves of flies infected with various concentrations of (A) the P. entomophila wild-type strain (Pe), (B) the mnl deficient strain (Δmnl), (C) the gacA-deficient strain (ΔgacA). UN = unchallenged. The number next to the bacterial strains indicates the concentration (Optical Density measured at 600nm) of the bacterial sample use for the infection. Survival assays have been performed at least three times in triplicate. (TIF) [file ppat.1002259.s006.tif]

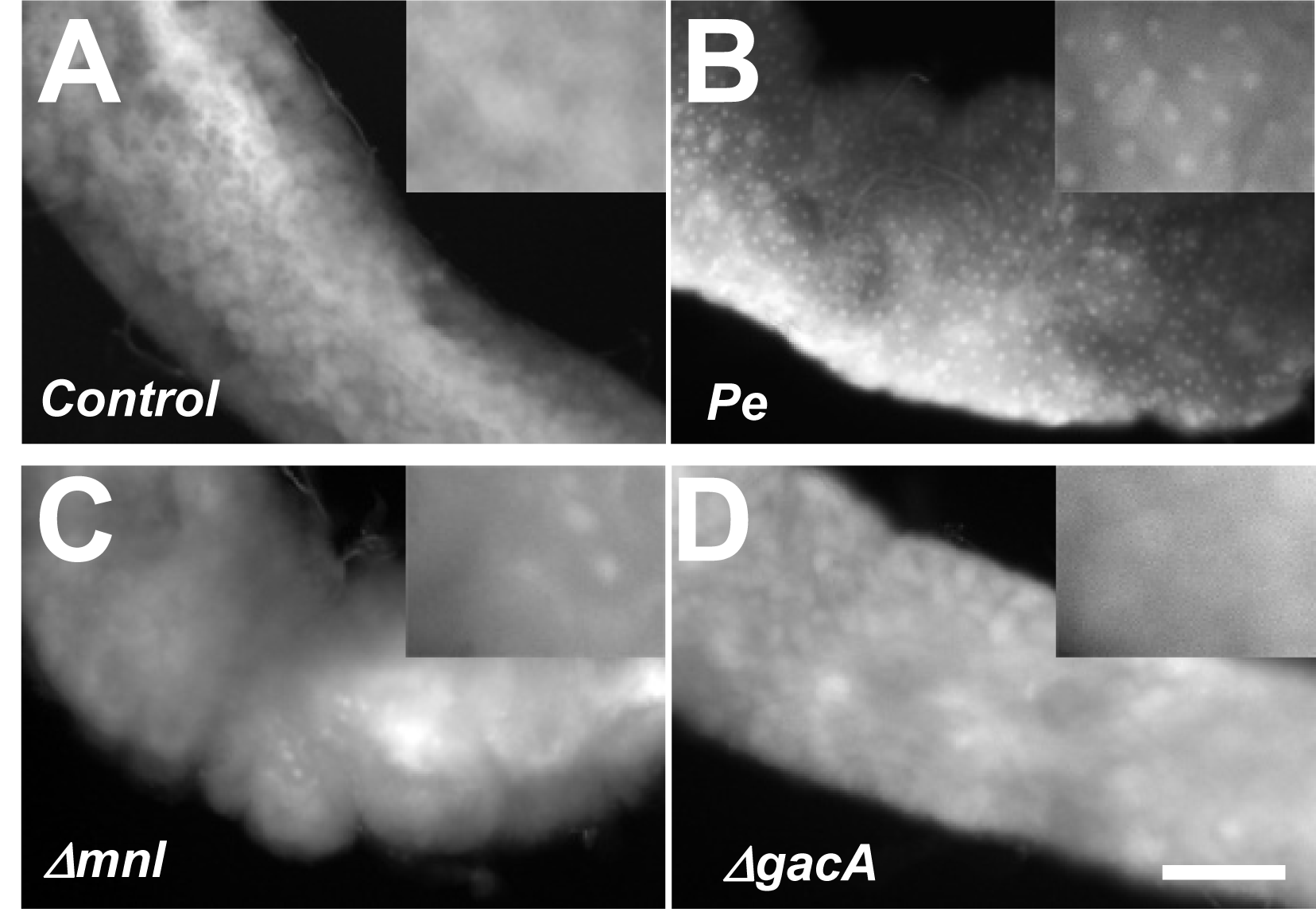

Supplement: Figure S7 — Cell death in guts of infected flies monitored by acridine orange staining. Guts were dissected from unchallenged female Oregon flies (A) or infected for 16 h with wild-type P. entomophila (B), a mnl mutant (C) or a gacA mutant (D), and stained with acridine orange. Scale bars represent 50 µm. (TIF) [file ppat.1002259.s007.tif]

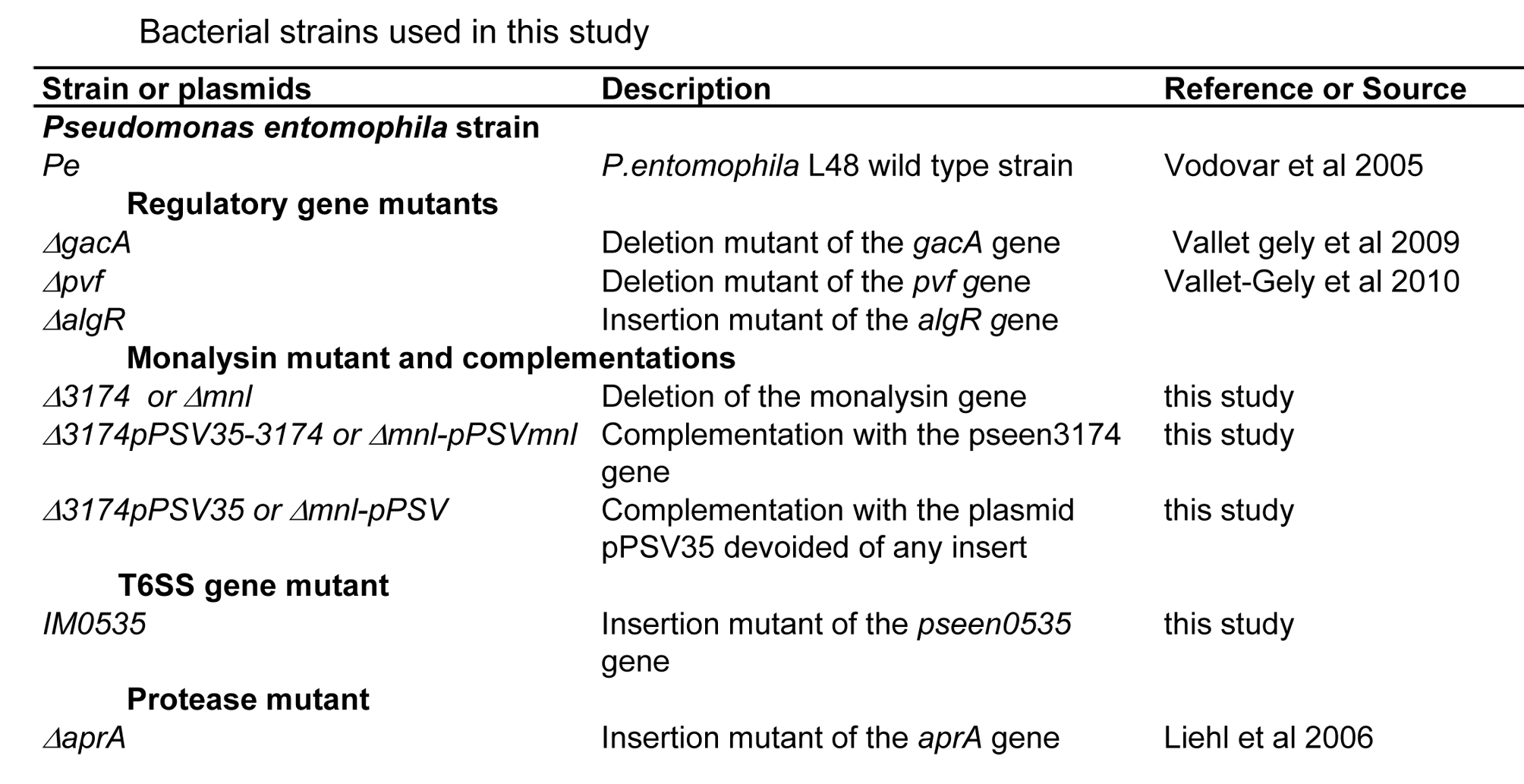

Supplement: Table S1 — Bacterial strains used in this study. (TIF) [file ppat.1002259.s008.tif]

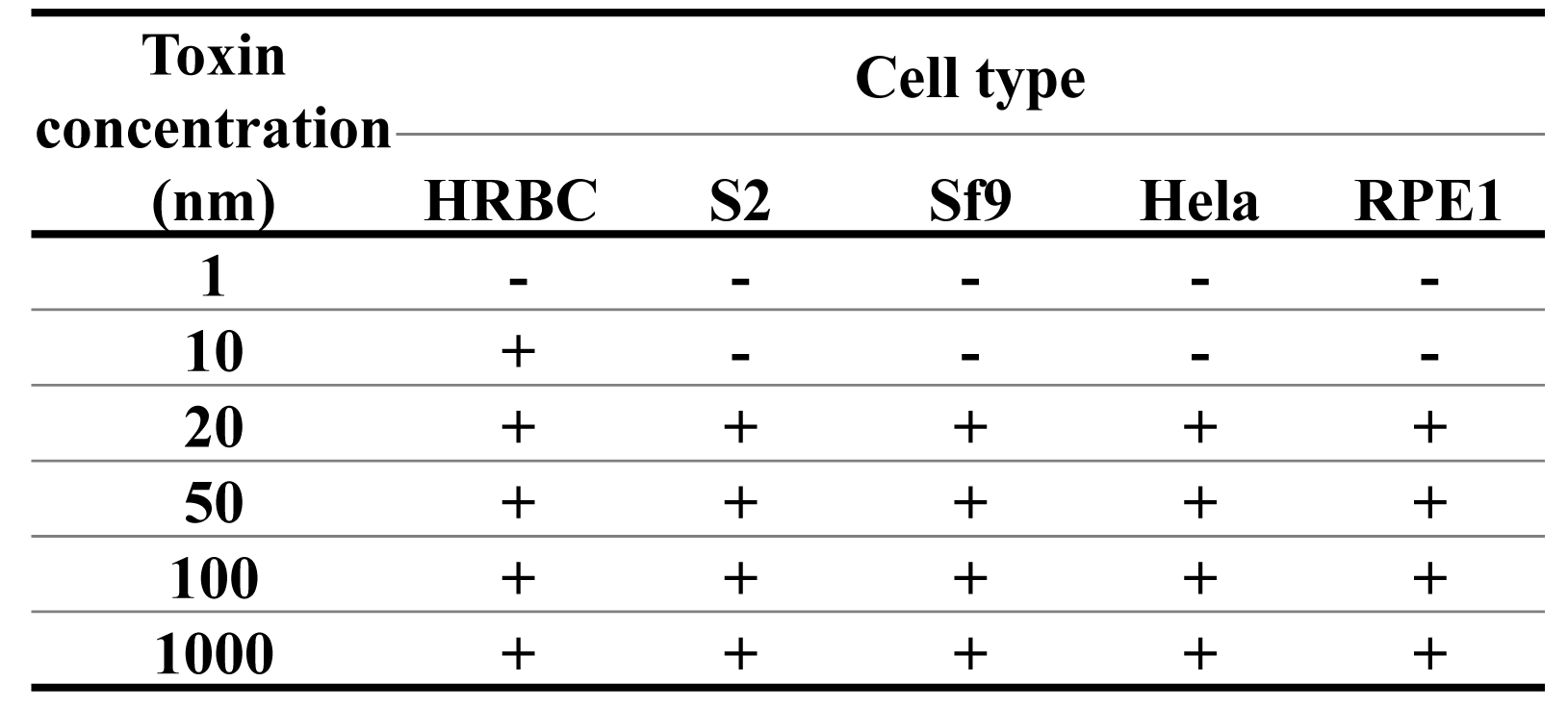

Supplement: Table S2 — Monalysin cytotoxicity towards insect and mammalian cells. Different culture cell lines were treated with the indicated concentration of Monalysin. Sensitivity (+) or resistance (−) to Monalysin was determined by phase contrast microscopy observation performed at 4 and 24 h. For HRBC (human red blood cells) the sensitivity was monitored by hemolytic activity as described in Material and Methods . (TIF) [file ppat.1002259.s009.tif]
